# Polio eradication in Africa: hard-won gains, prevailing challenges and what comes next

**DOI:** 10.11604/pamj.2026.53.38.48018

**Published:** 2026-01-27

**Authors:** Katherine Hayes, Kebba Touray, Nosheen Safdar, Jamal Ahmed

**Affiliations:** 1 Polio Eradication Programme, World Health Organization, Regional Office for Africa, Brazzaville, Republic of the Congo

**Keywords:** Public health, polio eradication, immunization

## Abstract

Polio eradication in Africa reached a significant milestone with the 2020 certification of the continent as wild poliovirus-free. However, the emergence of variant polioviruses continues to threaten progress, with 36 of 47 African countries reporting poliovirus detections since certification. This article discusses the ongoing challenges and strategic shifts required to eradicate all forms of poliovirus. This essay also discusses key breakthroughs in immunization strategies, such as the introduction of the novel oral polio vaccine and innovative tools like mobile money for frontline worker payments have advanced the eradication efforts. The path to full eradication remains complex, with a focus now on integrating polio efforts into broader health systems, addressing gaps in routine immunization and securing sustained funding.

## Essay

**Introduction:** polio may not dominate headlines worldwide, but its threat in Africa remains significant. When the continent was certified free of wild poliovirus in 2020, it was a major milestone in global health. But four years later, polio continues to paralyse children; however, this time around it is the variant strains. Since certification in August 2020 to December 2024, 36 of 47 countries in the WHO African Region have detected poliovirus, with reported cases of paralysis caused by both wild and variant polioviruses. This includes 9 cases of type 1 wild poliovirus that was imported in 2021, 1882 cases of type 2 variant poliovirus and 353 cases of type 1 variant poliovirus.

The resurgence of type 2 variant poliovirus has been a major challenge. Unlike wild poliovirus, this form emerges when an attenuated virus circulates and mutates in a population with very low immunity. While OPV remains a critical tool for immunization, low vaccination coverage and gaps in outbreak response have allowed the mutated virus to spread. Addressing this resurgence requires a shift in strategy, one that balances continued emergency response with long-term integration of polio efforts into routine immunization and broader health systems.

**Africa's ambitious polio eradication strategy:** the WHO African Region's eradication strategy for 2024-2025, *Road to Polio Eradication in Africa* [1], sets out a more aggressive approach to stopping all forms of poliovirus transmission. Unlike previous response strategies, which relied on a standard two-round campaign model, this plan adjusts outbreak response to risk levels, with affected countries conducting between three and five rounds as needed. Key elements of the strategy include reaching missed populations, strengthening surveillance in silent areas, improving vaccine allocation based on risk and tackling vaccine hesitancy through enhanced communication. Additionally, the plan emphasises integration with broader health services where feasible and securing renewed political and financial commitments. Its goals include closing wild poliovirus type 1 outbreaks by the end of 2024, which was met, and stopping variant type 1 outbreaks by the same deadline, which was not met. However, between August 2023 and August 2024, there has been a 96% reduction in type 1 variant poliovirus detections. The plan aims to end all ongoing type 2 variant poliovirus transmission by December 2025 and prevent further spread to new countries by 2026; however, the Global Polio Eradication Initiative (GPEI) extended timelines as part of its strategy revision in 2024 [2]. The revised timelines are to interrupt type 2 variant poliovirus transmission by 2026 and certify its elimination by the end of 2029.

**Breakthroughs and tools shaping polio eradication:** just two years after Africa was declared free of wild poliovirus in August 2020, wild poliovirus type 1 was detected once again on the continent. In November 2021, a case was reported from Malawi that could be traced back to Pakistan. Alarm bells rang across the region, and within months, additional cases surfaced in Mozambique. The risk of wild poliovirus transmission across the region was re-established. It was a stark reminder that until polio is wiped out everywhere, no country is safe. In response, a highly aggressive multi-country outbreak response was launched, including vaccination campaigns and enhanced surveillance. By August 2022, no further spread was detected. By May 2024, the outbreak was officially declared closed. This rapid containment of the wild poliovirus underscored the strength of Africa's eradication infrastructure when used aggressively, and the ever-present risk of resurgence.

The breakthroughs were enabled by core structures within the WHO Regional Office for Africa, including by the Regional Outbreak Response Group, which brings together GPEI partners to support countries in closing outbreaks effectively. Complementing this, the Rapid Response Team, including five subregional block leads and other experts, spearheads immediate case investigations, performs emergency coordination, ensures timely data flow and deploys technical experts to guide and mount outbreak response. Together, these two teams provide the rapid support needed to contain poliovirus outbreaks. Given the constant movement of people across the Region and the challenge of reaching some border communities with vaccination and surveillance, several cross-border coordination mechanisms were activated in 2024 to tackle the continued spread of poliovirus in Lake Chad Basin, Sahel and Horn of Africa countries. This was not the first time such an approach was needed. A similar mechanism for the Lake Chad Basin helped drive coordination until it was dismantled after the African Region was certified wild poliovirus-free. Alongside the coordination mechanisms, a joint plan for seven countries was developed (Burkina Faso, Cameroon, Central African Republic, Chad, Mali, Niger, and Nigeria), securing ministerial endorsement and setting out commitments for synchronised vaccination, data sharing and coordinated outbreak response. These efforts have helped countries work together rather than in isolation, making it easier to track the virus across borders, close immunity gaps and stop outbreaks before they spread further.

Another breakthrough was the rapid rollout of novel oral polio vaccine type 2 (nOPV2), making Africa the first region to deploy this next-generation vaccine to combat variant poliovirus outbreaks while aiming to reduce the risk of further mutations. Approved under WHO's Emergency Use Listing in late 2020, nOPV2 was introduced in Nigeria by March 2021, setting the stage for an unprecedented scale-up. In just a few years, over 1.1 trillion doses have been administered across more than 33 African countries, demonstrating the region's ability to rapidly adopt and integrate new tools into outbreak response. The vaccine has proven as effective as its predecessor in stopping outbreaks but with a lower risk of triggering new variant strains. Despite the logistical complexities of rolling out a vaccine with new requirements, African health systems adapted quickly.

Alongside advances in vaccination, technological innovations have played a critical role in closing the remaining gaps in Africa's polio response. One of the most transformative has been the use of mobile money, which replaced slow, unreliable cash payments with direct digital transfers to frontline workers. Between 2020-2024, over 2.3 million health workers across 23 countries have received over US $80 million in payments through this system, ensuring timely and transparent compensation that has strengthened workforce management and workforce retention. Meanwhile, the use of geographic information systems (GIS) and mobile health (mHealth) technologies to support surveillance and outbreak response activities has been pivotal in guiding polio eradication efforts in the African Region. Data tools like geospatial tracking systems (GTS), electronic surveillance, and environmental surveillance innovations have improved campaign quality, monitoring of surveillance activities and accountability of teams at different levels. GTS, deployed in seven priority countries so far, uses real-time location data to track areas covered or missed during vaccination outreach. It even works offline in areas without network coverage. The data, shown on maps or dashboards, helps supervisors quickly identify gaps and redirect teams. It also supports better microplanning and faster, more accurate monitoring and can store data for upload when offline, making it usable even without network access.

In the Democratic Republic of the Congo, for example, GTS was integrated into an enumeration exercise in between rounds in August 2023 following low geographic coverage in the Katuba health zone of Haut Katanga Province (65%). Following this, the geographic coverage improved to 82% in the subsequent round, demonstrating the value of GTS as part of the microplanning process. To support the scale-up and sustainable use of GIS innovations, GIS capacity-building efforts have also expanded. In 2022, three sub-regional trainings, conducted by Inter-Country Support Teams, trained 77 GIS focal points, data managers and surveillance officers across 21 countries. Building on these gains, the GIS Centre facilitated in-country trainings in Guinea (September 2023), Malawi (March 2024), and Madagascar (August 2024), equipping subnational staff with data visualisation and risk analysis skills to further strengthen surveillance and immunization planning.

eSURV, a mobile-based electronic surveillance tool, has also transformed disease surveillance, now deployed in 46/47 countries in the WHO African Region. It allows field officers to report real-time active surveillance data, thus improving oversight. As of November 2024, more than 500,000 eSURV visits were conducted, leading to the identification of a significant number of unreported, suspected cases of acute flaccid paralysis, COVID-19, neonatal tetanus, yellow fever and measles. In 2023, eSURV was further enhanced with the development of the map-centric Companion App, designed to assist countries to standardise and verify the development, prioritization and maintenance of their active surveillance sites using their mobile phones. It enables field officers' access to real-time information on the status of site visits, improving planning of supportive supervision activities. As of December 2024, seven countries have deployed the eSURV Companion App, with implementation support ongoing for others. Together, these innovations have not only advanced polio eradication efforts but also laid the foundation for stronger health systems across Africa, offering tools that can be applied to other disease programmes long after polio is gone.

**Prevailing challenges:** today, the greatest barriers to eradication are physical inaccessibility, operational challenges, vaccine hesitancy, weak health infrastructure and funding uncertainty. Countries like Nigeria, the Democratic Republic of the Congo and Chad are still experiencing huge outbreaks representing 74% of all reported types 1 and 2 for the period August 2020 to December 2024. The driving factors for this upward surge include challenges from conflicts and insecurity to systemic immunisation gaps in national health programmes. The polio programme itself cannot fill these immunization gaps alone. Its resources are already stretched as donor funding declines. A mix of factors has led to low immunization coverage and widening immunity gaps, particularly for different poliovirus serotypes.

The inactivated polio vaccine (IPV) is one of the major tools in the arsenal of polio eradication for the interruption of continued transmission of poliovirus in some of the consequential geographies, such as northwest Nigeria and eastern Democratic Republic of the Congo. Whilst the introduction of the first dose of the inactivated polio vaccine has now occurred in 47 countries in the Region, the second-dose initiation has been proved more challenging, with 21 countries yet to begin its introduction. Within the GPEI, funding shortfalls have forced difficult prioritization decisions, leading to postponed or cancelled polio immunization campaigns in at least 15 countries. Supply disruptions for nOPV2 have also complicated outbreak responses, delaying efforts to stop transmission.

Beyond funding constraints, operational challenges persist. Insecurity, political instability and humanitarian crises continue to hinder access to high-risk populations. Additionally, fake finger-marking, microplanning gaps and inefficiencies in executing quality campaigns have weakened vaccination efforts, leading to breakthrough cases and continued detections in environmental surveillance, even in areas that have conducted multiple rounds.

Another key challenge has been gaps in surveillance. Between 2023-2024, 112 orphan viruses were detected in 21 countries, indicating surveillance gaps at the subnational level. Through expansion efforts, all 47 countries in the African Region have now implemented environmental surveillance; however, many sites continue to underperform. Only 18 countries with functional environmental surveillance sites met the target of having 80% of sites achieve an enterovirus detection rate of 50% over 12 months in 2024. Many of the countries that continue to face outbreaks have chronically low routine immunization coverage, leaving large cohorts of under-immunized children vulnerable to infection.

Vaccine hesitancy remains another major obstacle, with refusal rates exceeding 15% in some urban areas, particularly due to misinformation, religious resistance and lack of trust in health authorities. This is especially concerning in places such as northern Nigeria, the Democratic Republic of the Congo and Ethiopia, where persistent refusals have hindered immunization delivery. In many high-risk areas, social mobilization and engagement strategies have been weak, with country programmes failing to sustain community outreach beyond polio campaigns. The greatest challenge ahead is closing immunization and surveillance gaps, ensuring that every child receives protection against all poliovirus types.

**What remains to sustain gains and certify eradication of variant polioviruses?** despite major breakthroughs in Africa's fight against polio, the path to lasting wild poliovirus eradication and achieving eradication of variant polioviruses remains complex. Ensuring highly sensitive surveillance systems and sustaining high routine immunization coverage beyond supplementary vaccination responses is essential if polio is to become the second human disease ever to be eradicated. However, the polio programme itself cannot fund this transformation alone. Its resources are already stretched, with donor commitments declining as eradication nears. But what it does have is expertise, infrastructure and a deep field presence and expertise in some of the most high-risk geographies.

The polio workforce has been one of the most extensive health delivery networks in Africa, with thousands of trained vaccinators, surveillance officers and data managers in polio-priority countries who could be leveraged to strengthen routine immunization systems. Polio teams are already supporting essential immunization efforts by integrating polio vaccines into measles and yellow fever campaigns, providing microplanning expertise to national routine immunization programmes and using data tools like GIS and mobile tracking to identify zero-dose children.

The GPEI's Independent Monitoring Board for Polio Eradication has emphasized the opportunity for stronger collaboration between the polio programme and partners such as Gavi, the Vaccine Alliance and the World Bank, which are funding routine immunization but lack the same operational reach [3]. The challenge now is to transition polio's operational strengths into long-term health system benefits, ensuring that Africa does not simply stop the spread of polio but builds resilience against future outbreaks of all vaccine-preventable diseases.

Funding uncertainty is another major obstacle. As eradication nears, donor support is declining. A premature drop in funding could leave countries unable to maintain high-quality surveillance and strong outbreak response capacity, opening the door for a resurgence. This is particularly concerning given that wild poliovirus type 1 continues to circulate in Afghanistan and Pakistan. Cross-border collaboration and sustained investment in high-risk areas remain critical to ensuring circulating variant polioviruses are eliminated and the wild poliovirus does not return to Africa.

Finally, declaring the African Region polio-free requires several years without a single poliovirus detection in the presence of strong surveillance systems. This requires strengthening environmental surveillance, rapid AFP case detection, better integration with existing systems and stronger national ownership of polio oversight. After official eradication, post-certification strategies must be in place to prevent resurgence. The ultimate test is not just to end polio transmission, but to ensure that it never returns.
